# Acetylcholine Receptor Stimulation Activates Protein Kinase C Mediated Internalization of the Dopamine Transporter

**DOI:** 10.3389/fncel.2021.662216

**Published:** 2021-04-09

**Authors:** Suzanne M. Underhill, Susan G. Amara

**Affiliations:** National Institute of Mental Health, National Institutes of Health (NIH), Bethesda, MD, United States

**Keywords:** dopamine transporter, muscarinic receptor, trafficking, protein kinase C, internalization

## Abstract

The dopamine transporter (DAT) clears neurotransmitters from the extracellular space and serves as an important regulator of signal amplitude and duration at sites of dopamine release. Several different intracellular signaling pathways have been observed to modulate DAT activity through the regulation of the trafficking of the carriers to and from the cell surface. Acute activation of protein kinase C (PKC) by phorbol esters facilitates clathrin-dependent internalization of the DAT in a variety of model systems; however, the physiological stimuli and cell-surface receptor systems that activate PKC and regulate the DAT in dopamine neurons remain elusive. We report here that stimulation of M_1_/M_5_ muscarinic receptors in midbrain cultures decreases the ability of dopamine neurons to transport dopamine through DAT. Application of the cholinomimetic drug carbachol leads to a decrease in DAT activity in primary cultures while the M_1_/M_5_-specific antagonist, pirenzepine, blocks these effects. The M_3_ antagonist, DAU 5884, does not affect, but a positive modulator of M_5_, VU 0238429, enhances the loss of DAT function in response to carbachol and acetylcholine. These data implicate M_1_/M_5_ receptors on dopamine neurons in the modulation of DAT function. Bisindolylmaleimide, a PKC inhibitor, blocks the effects of carbachol stimulation on dopamine uptake, supporting a role for PKC in muscarinic receptor-mediated DAT internalization. Furthermore, as shown previously for PKC-induced internalization, downregulation of the DAT is dependent on both clathrin and dynamin. A G_q_-specific inhibitor peptide also blocks the effects of carbachol on DAT in primary cultures, confirming G_q_ as the G-protein that couples M_1_/M_5_ receptors to PKC activation in these cells. In acute midbrain slices, biotinylation of cell-surface proteins revealed the loss of dopamine transport mediated by muscarinic receptor stimulation was, indeed, due to loss of membrane expression of the DAT in endogenous tissue. These data indicate that stimulation of cholinergic pathways can lead to modulation of dopamine through internalization of the DAT.

## Introduction

The dopamine transporter (DAT) clears neurotransmitters from the extracellular space and serves as an important regulator of signal amplitude and duration at sites of dopamine release. In both dopamine (DA) neurons and transfected cell lines, the activation of several different intracellular signaling pathways has been observed to modulate DAT activity by regulating the trafficking of carriers to and from the cell surface (Mortensen and Amara, 2003). Protein kinase C (PKC) signaling has been extensively studied as a mechanism for regulating transporter cell surface density, and it has been well-established that acute activation of PKC by phorbol esters facilitates internalization of the transporter in a variety of model systems. However, the physiological stimuli and cell systems that activate PKC and potentially regulate the DAT in dopamine neurons within the mammalian brain remain elusive.

There are several G-protein coupled receptors (GPCRs) that may be coupled to cascades that lead to PKC-activation that are present on dopamine neurons, several of which belong to the muscarinic acetylcholine receptor family. Acetylcholine receptors fall into two broad categories, nicotinic and muscarinic receptors. Nicotinic receptors are ligand-gated ion channels while muscarinic receptors are GPCRs. Of the five isoforms of muscarinic receptors, M_1_, M_3_, and M_5_ are G_Q/11_-coupled while M_2_ and M_4_ are G_i_-coupled. G_Q/11_-coupled GPCRs may lead to PKC activation and suggest a mechanism by which DAT may be modulated through PKC in dopamine neurons through stimulation of M_1_, M_3_, and/or M_5_ receptors.

In this study, we explored this question and further characterized the intracellular mechanism of DAT internalization in response to acetylcholine receptor stimulation in dopamine neurons. DAT internalization has been well described in response to both amphetamine (Kahlig et al., 2006; Hong and Amara, 2013; Wheeler et al., 2015) and phorbol esters, such as PMA (Vaughan et al., 1997; Zhu et al., 1997; Daniels and Amara, 1999; Melikian and Buckley, 1999). However, internalization of the DAT transporter by amphetamine or PMA occurs through distinct cellular pathways, the former being mediated by a clathrin-independent and RhoA-dependent process while the latter is explicitly clathrin-dependent (Daniels and Amara, 1999; Hong and Amara, 2013; Wu et al., 2015). This report addresses the mechanism of DAT internalization in response to muscarinic receptor stimulation in dopamine neurons.

## Materials and Methods

### Primary MidBrain Cultures

**Figure 1:**
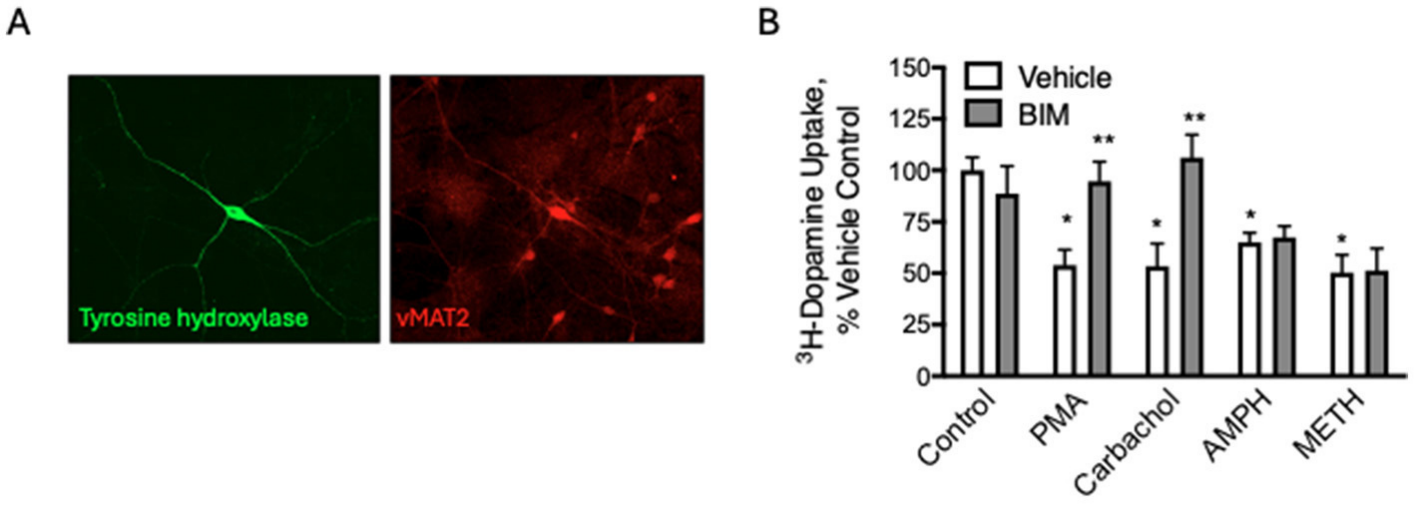
In primary midbrain cultures, PMA and carbachol-mediated loss of DAT function are PKC mediated while AMPH and METH-mediated redistribution are not. **(A)** Primary mid-brain cultures have dopamine neurons that express tyrosine hydroxylase (TH) and the vesicular monoamine transporter 2 (vMAT2). **(B)** Pre-treatment of these cultures with PMA (10 μM), carbachol (30 μM), amphetamine (10 μM, AMPH) and methamphetamine (10 μM METH) all decreased transport capacity of DAT (defined as GBR-12909 sensitive ^3^H-dopamine uptake). Bisindolylmaleimide I (BIM, 1 μM) inhibited the effects of PMA and carbachol, substantiating a PKC-mediated effect on DAT by these agonists. BIM did not alter the loss of DAT activity mediated by pretreatment with AMPH or METH indicating independent mechanisms of DAT internalization (*n* = 3).

All procedures using animals were conducted in compliance with protocols approved by the ACUC at the National Institutes of Health (NIH). Primary cultures were obtained as described previously (Wheeler et al., 2015) from midbrains of E15 Swiss-Webster mice following isofluorane anesthesia and cervical dislocation of the pregnant dam. The tissue was gently triturated, plated at a density of 0.16 midbrains per 12-mm poly-D-lysine coated coverslips or 0.33 per 25-mm coverslip. Cells were grown in Modified Eagle Medium (MEM) supplemented with 5% horse serum and 5% fetal calf serum for 2–5 weeks to facilitate the formation of synapses and expression of DAT, vMAT, and M_1_/M_5_ (Figures 1, 3). Neuromag was used to transfect primary neurons according to the manufacturer’s directions (OZ Biosciences, NM50200).

**Figure 2:**
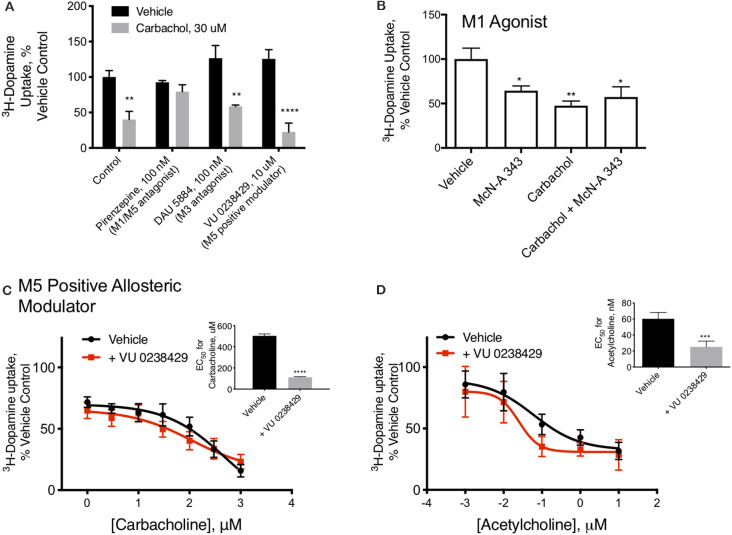
M_1_/M_5_ receptors mediate DAT redistribution. **(A)** In primary midbrain cultures, the effect of carbachol pretreatment on DAT-mediated dopamine transport were was blocked by the M_1_/M_5_ antagonist pirenzepine (100 μM). The M_3_ antagonist, DAU 5884 (1 μM), had no effect ondid not affect the activity of the DAT. The positive M_5_ modulator VU 0238429 (10 μM) potentiated the effects of carbachol (*n* = 6, 6, 4, 3, 5, 4, 6, 4, **P ≤ 0.01 and ****P ≤ 0.0001 by two-way ANOVA). **(B)** Pretreatment of primary midbrain cultures with the M_1_/M_5_ agonist, McN-A-343 (10 μM), decreased DAT-mediated dopamine transport. McN-A-343 did not demonstrate an additive effect with carbachol, suggesting they are both affecting DAT localization through muscarinic receptor activation (*n* = 4, *P ≤ 0.05 and **P ≤ 0.01 by one-way ANOVA). **(C)** Co-application of the M_5_-positive modulator VU 0238429 potentiates the effects of carbachol in primary midbrain cultures (*n* = 7, ***P ≤ 0.001 by *t*-test). **(D)** Acetylcholine-mediated internalization of DAT in primary cultures is also enhanced by co-application of VU 0238429 (*n* = 4, ***P ≤ 0.001 by *t*-test).

**Figure 3:**
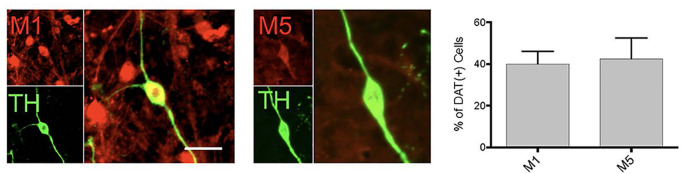
DA neurons in primary midbrain culture also express muscarinic acetylcholine receptors. Cultured TH( + ) (green) neurons were found to also express M_1_ and M_5_ receptors (red). Quantitation of the number of TH( + ) neurons that also expressed M_1_ or M_5_ indicated that 40% of TH neurons expression expressed M_1_ and 42.5% express M_5_ [*n* = 11 fields of view, 155 and 112 TH( + ) cells assessed for M_1_ or M_5_ co-expression, respectively; scale bar 25 μm].

### ^3^H-Dopamine Transport Assays

Cells were exposed to vehicle or drug treatments for 30 min at 37° in MEM, without serum. Cells were then washed and dopamine transport was determined by a 7-min incubation with 50 nM ^3^H-dopamine and 20 μM cold dopamine in phosphate-buffered saline (PBS) supplemented with 1 mM calcium chloride and 100 μM magnesium chloride. Cells were washed with PBS, lysed with scintillation fluid, and the cpm obtained on a Beckman scintillation counter. Control samples treated with the DAT-specific antagonist GBR-12909 (10 nM, Tocris, 0421) were used to determine non-specific background radiation in primary cultures. Data are presented with the background-subtracted and normalized to vehicle-treated controls. Assays were performed on at least three different occasions from three different culture-dates. Vehicle control values were at least 2-fold above the background determined by inhibition of DAT-specific uptake with GBR-12909.

### Biotinylation of Acute Brain Slices

Cell-surface proteins in acute brain slices was performed as described previously (Underhill et al., 2014). Briefly, 12–24 week-old Swiss-Webster mice were anesthetized with isofluorane, euthanized by cervical dislocation, and decapitated. Brains were isolated and 1-mm mid-brain sections were cut with a brain slicer matrix in ice-cold Hank’s Buffered Saline Solution. Slices were transferred to 1 ml of artificial cerebrospinal fluid (aCSF, in mM: 126 NaCl, 2.5 KCl, 2.4 CaCl_2_, 1.2 MgCl_2_, 1.2 NaH_2_PO_4_, 21.4 NaHCO_3_, 11.1 D-Dextrose, pH 7.4) with 50 μM kynurenic acid (Sigma, K3375) to prevent excitotoxic cell damage and allowed to rest for 30–60 min at room temperature. Treatments of slices were performed in 1 ml of aCSF bubbled with 95% oxygen and 5% carbon dioxide at 37°C in a water bath for 30 min, chilled to 4°C on ice for 10 min, and incubated in NHS-SS-biotin (Thermo Fisher, 21331) in biotinylation buffer (in mM: 2 CaCl_2_, 150 NaCl, and 10 triethanolamine, pH 7.5) for 20 min at 4°C on ice. The biotin reagent was quenched with 50 mM glycine for 10 min and then washed off with PBS. Tissue was lysed in 1 ml 1% triton buffer and dounce homogenized. Nuclear fractions were removed by centrifugation for 20 min at 15,000 rpm at 4°C. Biotinylated proteins were isolated by incubation overnight at 4°C with Ultralink immobilized Pierce NeutrAvidin beads (Thermo Fisher, 29200) and analyzed by western blot.

### Western Blots

Protein samples were loaded in Tris-Glycine gels (4–12%, Thermo Fisher, SV04125) in Laemmi’s buffer supplemented with 200 mM DTT. Gels were transferred to PVDF membrane, blocked with 5% milk, and probed with primary antibodies (1:1,000) overnight. Membranes were washed with PBS-Tween and secondary antibodies (HRP-conjugated donkey anti-rabbit, 1:10,000) were applied for 1 h in 5% milk and washed with PBS-tween. Probes were imaged with chemiluminescence and quantified only in non-saturating images by ImageJ. Data were adjusted for total input from lysate samples taken before NeutrAvidin isolation of biotinylated proteins. Total inputs did not vary across these assays consistent with equal protein concentrations and sample loading across preparations.

### Immunocytochemistry

Cultured cells were fixed with 4% paraformaldehyde at 4°C for 20 min and washed with PBS. Cells were permeablized with a 5-min treatment with 0.25% Triton X-100 and then blocked with 5% normal goat serum (NGS) for 1 h at room temperature. Primary antibodies were diluted in 5% NGS were incubated on the cells overnight at 4°C. Secondary antibodies were applied for 1 h at room temperature in 5% NGS at room temperature. Cultures were then washed with PBS and mounted in AntiFade reagent. Images were acquired with a Nikon Axiovert confocal microscope.

### Reagents

Primary antibodies included rabbit anti-M_1_ (Abcam, ab111100), rabbit anti-M_5_ (Abcam, ab41171), chicken anti-tyrosine hydroxylase (Aves, TYH), rat anti-DAT (Thermo Fisher, mAb16), and rabbit anti-vMAT (Millipore, AB1598P). HRP, Dylight-488, or Alexa 568 conjugated secondary antibodies were from Jackson Immunoresearch. Dynole 34-2 and Pitstop 2 and the negative control were both from Abcam. Custom-designed TAT-peptides were ordered from LifeTein.

### Quantitation and Statistics

All cell culture assays were performed on cultures from at least three separate preparations. For ^3^H_DA uptake assays, one coverslip was used as an *n* of 1. M_1_/M_5_ immunolabeling was manually counted over 11 60× fields of view from three different culture dates. One-hundred and fifty-five and 112 TH( + ) cells were assessed for M_1_ or M_5_ co-expression, respectively. Clathrin and DAT colocalization was quantified by ImageJ using sub-threshold images. All statistics were performed with GraphPad Prism software. *P ≤ 0.05, **P ≤ 0.01, ***P ≤ 0.001 and ****P ≤ 0.0001 throughout the manuscript for one-way ANOVA, two-way ANOVA or *t*-test evaluations.

## Results

Dopamine transport in neurons is modified by stimulation of muscarinic acetylcholine (Ach) receptors. Dopamine neurons were cultivated from E15 mouse mid-brains. Dopamine neurons in these cultures expressed DAT, tyrosine hydroxylase (TH), and the vesicular monoamine transporter 2 (vMAT2; Figure 1A), as expected for well-differentiated DA neurons in culture. DAT transport activity was assessed in these cultures with ^3^H-dopamine and defined as that which was sensitive to the DAT-specific inhibitor GBR12909. We found that a 30-min pre-treatment of these cultures with phorbol 12-myristate 13-acetate (PMA, 10 μM) decreased subsequent GBR12909-sensitive, DAT-mediated transport of ^3^H-dopamine in these cultures (Figure 1B). This effect was blocked by co-application of the PKC inhibitor bisindolylmaleimide (BIM, 1 μM) 5 min before and concurrent with PMA pretreatment, indicating that the loss of DAT transport activity depends on PKC activation. Similarly, we found that pre-treatment with the muscarinic receptor agonist carbachol (30 μM) could also decrease dopamine transport. The effects of carbachol pre-treatment on DAT transport were also inhibited by BIM. These data indicate that muscarinic Ach receptors can stimulate PKC to affect DAT function in DA neurons. Amphetamine and methamphetamine pretreatments also decreased DAT transport capacity in these midbrain neurons, however, these effects were not altered by PKC inhibition by BIM.

M_1_/M_5_ muscarinic receptors alter DAT function. Carbachol is known to stimulate all five of the muscarinic GPCRs. Isoforms 1, 3, and 5 are G_q_/11 coupled, suggesting a mechanism by which carbachol could stimulate PKC, while isoforms 2 and 4 are G_i_-coupled. We tested antagonists to isoforms M_1_/M_5_ and M_3_ in combination with carbachol. The M_1_/M_5_ antagonist pirenzepine blocked the effects of carbachol on dopamine transport (Figure 2A). The M_3_ antagonist DAU5884 did not affect while the M_5_ positive allosteric modulator VU 0238429 potentiated the effect of carbachol and acetylcholine on DAT activity (Figures 2A,C,D). The insets indicate the EC50 values for carbachol (Figure 2C) or acetylcholine (Figure 2D) with and without VU 0238429 to perform the statistical evaluation. Further, the M_1_/M_5_ receptor agonist McN-A-343 mimicked the effects of carbachol in decreasing DAT transport capacity, supporting the role of M_1_/M_5_ receptor stimulation in DAT modulation (Figure 2B).

Consistent with the pharmacological profile indicating that the DA neurons in primary culture can be modulated by M_1_ and M_5_ receptors, we found expression of both of these receptors in DA neurons (Figure 3). However, only ~40% of the dopamine neurons expressed these receptors, indicating a population of DA neurons would not be affected by muscarinic receptor agonists. With the antibodies currently available, we were not able to further characterize M_1_/M_5_ expression in the same cells.

**Figure 4:**
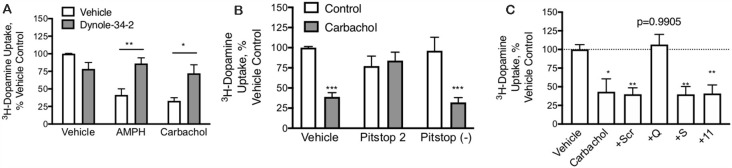
DAT-mediated internalization by carbachol is dependent on both dynamin and clathrin. **(A)** In primary midbrain cultures, dynole (50 μM), an inhibitor of dynamin, prevented DAT internalization in response to both AMPH (10 μM) and carbachol (30 μM). These data further support the internalization of the transporter as the mechanism of loss of DAT function (*n* = 3, *P ≤ 0.05 and **P ≤ 0.01 by two-way ANOVA). **(B)** Pitstop II (30 μM), a clathrin inhibitor, prevents DAT internalization in response to carbachol. There was no affect effect of this clathrin inhibition on AMPH-mediated DAT internalization, consistent with prior studies demonstrating this as a clathrin-independent process (*n* = 3, ***P ≤ 0.001 by two-way ANOVA). **(C)** TAT-fused peptides that were designed to interfere with GPCRs coupled to G_Q_, G_S_, or G_11_ alpha subunits were applied to dopamine neurons in primary culture (70 μM). Subsequent treatment of these cultures with carbachol led to the loss of DAT-mediated DA transport under all conditions, except the TAT-G_Q_ pre-treated cultures (*n* = 5, *P ≤ 0.05 and **P ≤ 0.01 by one-way ANOVA).

Mechanism of DAT Regulation by M_1_/M_5_ Receptors PMA/PKC stimulated internalization of DAT is a dynamin (Gabriel et al., 2013) and clathrin-dependent (Daniels and Amara, 1999) process whereas AMPH-mediated DAT internalization is also dynamin-dependent (Saunders et al., 2000) but is clathrin-independent (Wheeler et al., 2015). In primary midbrain cultures, both carbachol and amphetamine produced a loss of DAT activity that was blocked by the dynamin inhibitor dynole 34-2 (Figure 4A), confirming that the effects of both drugs depend on dynamin. Inhibition of the formation of clathrin-coated pits by Pitstop 2 also blocked the downregulation of the DAT by carbachol (Figure 4B) demonstrating that the process depends on clathrin. The negative control compound for Pitstop 2 did not have any effect on carbachol-mediated DAT internalization.

M_1_ and M_5_ receptors are G_Q_-coupled. The G_Q_ μ-subunit of G-proteins signals by increasing inositol triphosphate (IP3) and diacylglycerol (DAG), which acts as a second messenger to mobilize intracellular calcium stores and activate PKC. We designed short peptides based on the C-terminus of various GPCR α-subunits that we have shown interfere selectively with their respective signaling pathways (Underhill et al., 2019). These peptides were made cell-permeable with the addition of a short TAT domain (YGRKKRRQRRR), as described previously (Underhill et al., 2019). Interfering with G_S_ or G_11_ subunits did not affect the carbachol-mediated loss of DAT uptake; however, the G_Q_ interfering peptide could completely abolish the effect (Figure 4C).

**Figure 5:**
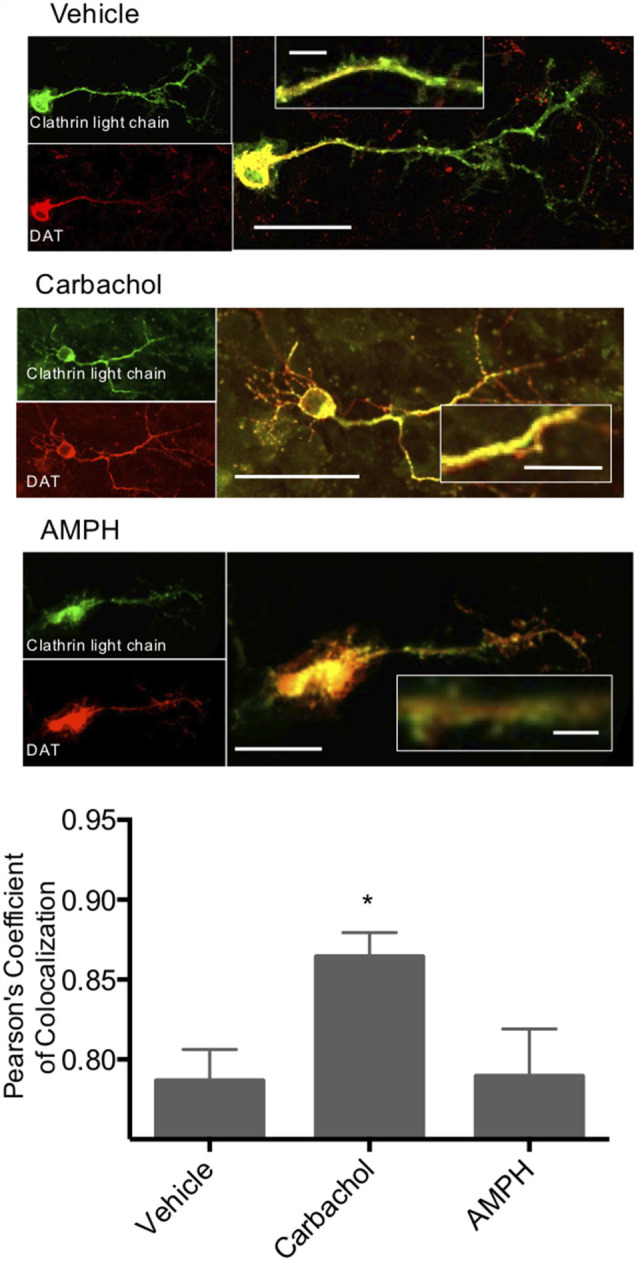
Clathrin and DAT colocalize in response to carbachol. Primary midbrain neurons were transfected with a GFP-tagged clathrin light chain and an mCherry-tagged DAT. Colocalization of these two proteins was significantly increased in response to carbachol treatment (30 μM). Treatment of the cultures with amphetamine did not alter co-localization. *Indicates *p* < 0.05 by one-way ANOVA. *n* = 19, 15, and 12, respectively. Scale *bars* = 50 μm for merged images, 10 μm for the insets.

To further investigate the role of clathrin in M_1_/M_5_ stimulated internalization of DAT, we coexpressed GFP-tagged clathrin light chain and mCherry-tagged DAT in primary cultured neurons. These cells were then treated with vehicle control, carbachol, or amphetamine, and we measured the colocalization of the DAT with clathrin in response to these stimuli (Figure 5). Carbachol treatment enhanced the colocalization of the transporter with the coat protein, clathrin.

Carbachol treatment causes clathrin-mediated DAT internalization. To further investigate the role of clathrin in M_1_/M_5_ stimulated internalization of DAT, we coexpressed GFP-tagged clathrin light chain and mCherry-tagged DAT in primary cultured neurons. These cells were then treated with vehicle control, carbachol, or amphetamine, and we measured the colocalization of the DAT with clathrin in response to these stimuli (Figure 5). Carbachol treatment enhanced the colocalization of the transporter with the vesicle coat protein, clathrin.

**Figure 6:**
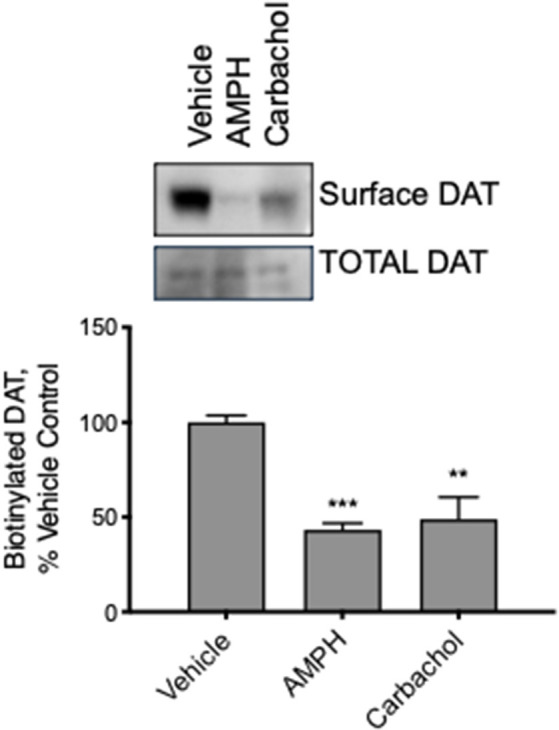
DAT internalizes in response to AMPH and carbachol in acute brain slices from adult mice. We exposed slices from mature mouse brain to vehicle, AMPH or carbachol for 30 min and then biotinylated all the surface proteins, isolated the biotinylated proteins, and assessed DAT localization by Western blot. Carbachol (30 μM) as well as AMPH (10 μM), as well as AMPH (10 M), decreased the amount of biotinylated, membrane membrane-localized DAT (*n* = 3, **P ≤ 0.01 and ***P ≤ 0.001 by one-way ANOVA).

Modulation of DAT transport function may result from several actions on the DAT; however, we hypothesized that muscarinic receptor stimulation causes loss of DAT function through the trafficking of the carrier away from the plasma membrane through a mechanism similar to that observed with PKC activation by PMA. We treated mid-brain acute slices from adult mice with vehicle, amphetamine, or carbachol. Subsequently, all surface proteins were labeled with a cell-impermeable biotin reagent. The tissue was lysed, biotinylated proteins isolated, and analyzed by western blot. Membrane localized, biotin accessible DAT was significantly decreased in tissues treated with amphetamine or carbachol (Figure 6). These results were observed in both male and female female-derived tissues and we did not detect any difference across the groups.

## Discussion

The regulated internalization of the dopamine transporter contributes to increases in extracellular dopamine. Efforts have focused on two major endocytic mechanisms for the DAT: amphetamine-mediated internalization which depends on dynamin and the activation of another GTPase, RhoA (Wheeler et al., 2015), and PKC-mediated internalization which depends on dynamin and the formation of clathrin-coated pits (Daniels and Amara, 1999). Although several GPCRs linked to PKC activation could potentially regulate the DAT, muscarinic M_5_ receptors are strong candidates for several reasons. M_5_ GPCRs are expressed by midbrain DA neurons, are coupled to PKC activation, and have been shown to produce prolonged facilitation of dopamine release* in vivo* (Forster et al., 2002; Foster et al., 2014). In the current study, we show here that stimulation of the M_1_/M_5_ muscarinic acetylcholine receptors on DA neurons leads to activation of PKC and endocytosis of DAT, a series of events that could contribute to the elevations in extracellular DA concentrations observed *in vivo*.

Mechanism of M_1_/M_5_-mediated internalization. Previous studies have used the phorbol ester PMA to stimulate PKC and observe the internalization of DAT (Doolen and Zahniser, 2002). M_1_/M_5_ muscarinic acetylcholine receptors are G_Q_ coupled, resulting in phospholipase C activation, DAG release, Ca^2+^-release, and activation of PKC. We report here that M_1_/M_5_-receptor receptor-stimulated PKC activation is sufficient to internalize DAT in dopamine neurons. The mechanism of PKC-mediated internalization of DAT is dependent on dynamin and clathrin (Daniels and Amara, 1999), similar to what we report here for the action of carbachol.

PKC activation by PMA leads to ubiquitination of the transporter (Miranda et al., 2005). DAT is then internalized and sent towards a degradation pathway within 2 h of PKC activation (Daniels and Amara, 1999; Mortensen et al., 2008; Hong and Amara, 2013). It is unclear whether M_1_/M_5_-stimulated DAT internalization follows the same fate. Other studies have implicated the Ras-like GTPase Rin (Navaroli et al., 2011) and CDC42-activated, nonreceptor tyrosine kinase, Ack1 (Wu et al., 2015) to be involved in PKC-mediated DAT trafficking. How the various pathways and regulatory molecules are distributed across different subcellular compartments and anatomical regions will determine the potential impact of M_1_/M_5_-mediated internalization of the DAT on dopamine signaling. Understanding the precise localization of PKC-coupled GPCRs relative to the DAT is critical for establishing the physiological impact of PKC-regulated DAT trafficking. We found M_1_/M_5_ receptor stimulation of DAT(+) neurons in culture and acute brain slices was sufficient to modulate DAT membrane localization indicating the M_1_/M_5_ receptors are near DAT in DA neurons—whether, in axons, dendrites or discrete subcellular membrane domains remains to be further investigated.

In the absence of a physiological ligand that signals through PKC, the relevance of DAT internalization associated with the direct activation of PKC by PMA has remained unresolved. Moreover, PMA effects have not been observed consistently in cultured midbrain DA neurons. For example, one study that used a fluorescently cocaine-analog to probe trafficking of endogenous DAT in cultured midbrain neurons showed no evidence of PMA-induced internalization (Eriksen et al., 2009). The absence of an effect could have several explanations including off-target effects of PMA, which activates a broad range of PKC isoforms or an altered conformation of the ligand-bound carrier that precludes PMA-induced endocytosis. In support of this latter possibility, the Vaughan lab has reported that the DAT inhibitor GBR12909 blocked PMA-mediated phosphorylation of DAT indicating some that the conformational state of DAT can alter PKC-mediated effects on the carrier (Gorentla and Vaughan, 2005). To avoid the potential confounds associated with using PMA in brain preparations we have used a more physiologically-relevant endogenous receptor to activate PKC, Our results show that in primary DA neuron cultures and acute brain slices, M_1_/M_5_ receptor stimulation produces a robust downregulation of the DAT through a G_q_-coupled pathway, indicating that the endogenous DAT is responsive to GPCR-mediated PKC activation.

Interestingly, the membrane trafficking of several other neurotransmitter transporters is also affected by PKC activation. The norepinephrine transporter, NET, is internalized in response to methacholine in SKNSH cells (Apparsundaram et al., 1998a), and PMA in HEK-293, LLC-PK1, and COS-7 cells (Apparsundaram et al., 1998b). Expression of M_1_/M_5_ receptors in norepinephrine neurons or other G_Q_-coupled GPCRs suggest a physiological stimulus that may modulate norepinephrine signaling. The GABA transporter, GAT1, is also redistributed in response to PKC, mediated by acetylcholine, glutamate, and serotonin receptors (Beckman et al., 1999). We have found that PMA triggers internalization of the glutamate transporters EAAT1, EAAT2a (Susarla and Robinson, 2008), and EAAT2b (data not shown). Interestingly, PKC activation through PMA or platelet-derived growth factor (PDGF) stimulation leads to an increase in EAAT3 membrane localization several models (Gonzalez et al., 2003; Fournier et al., 2004). M_1_/M_5_ stimulation could contribute to EAAT3 trafficking that might be particularly important in GABA-ergic neurons where EAAT3 provides glutamate to these cells as a precursor to GABA synthesis. One report suggests that PMA can increase EAAT4 chloride currents that may be in response to PKC-induced regulation of that transporter (Fang et al., 2006). However, there was no measurable change in glutamate uptake, suggesting that the change in EAAT4 function may not be due to the altered trafficking of the carrier. Modulation of plasma membrane neurotransmitter transporters through G_Q_-coupled receptors, such as M_1_/M_5_ may play critical roles in neurotransmitter tone throughout the brain.

Circuits affected by M_1_/M_5_-mediated trafficking of DAT. M_1_/M_5_ receptors have been implicated in the mental decline in Alzheimer’s and Parkinson’s diseases as well as schizophrenia, all of which have been linked to various degrees of dysregulation in dopamine neurons (Conn et al., 2009). There is a large body of evidence that cholinergic inputs to the midbrain and basal ganglia circuitry modulate the activity and release properties of dopamine neurons (Forster et al., 2002; Threlfell et al., 2010, 2012; Foster et al., 2014). The phenomena that we describe here of M_1_/M_5_ receptor stimulation internalizing DAT indicates a precise mechanism by which acetylcholine can modulate dopamine neurons and extracellular dopamine concentrations directly. Targeting this particular pathway with the further development and exploration of drugs targeted to the M_1_/M_5_ receptor may lead to the development of better therapeutics.

## Data Availability Statement

The original contributions presented in the study are included in the article, further inquiries can be directed to the corresponding author.

## Ethics Statement

The animal study was reviewed and approved by NIMH Animal Care Committee approved under protocol #LMCN-1.

## Author Contributions

SU contributed to the conception and design of the study, performed the experiments, analyzed the data, and wrote the first draft and revisions of the manuscript. SA contributed to the conception and design of the study, manuscript revisions and approved the submitted version.

All authors contributed to the article and approved the submitted version.

## Conflict of Interest

The authors declare that the research was conducted in the absence of any commercial or financial relationships that could be construed as a potential conflict of interest.

## Correction Note

A correction has been made to this article. Details can be found at: 10.3389/fncel.2026.1864769.
