# Correction: Acetylcholine receptor stimulation activates protein kinase C mediated internalization of the dopamine transporter

**DOI:** 10.3389/fncel.2026.1864769

**Published:** 2026-06-17

**Authors:** Suzanne M. Underhill, Susan G. Amara

**Affiliations:** National Institute of Mental Health, National Institutes of Health (NIH), Bethesda, MD, United States

**Keywords:** dopamine transporter, muscarinic receptor, trafficking, protein kinase C, internalization

There was a mistake in Figure 1A as published. The first two panels exhibiting TH and DAT immunolabeling had been used in a prior publication. The corrected figure and legend appears below.

**Figure 1:**
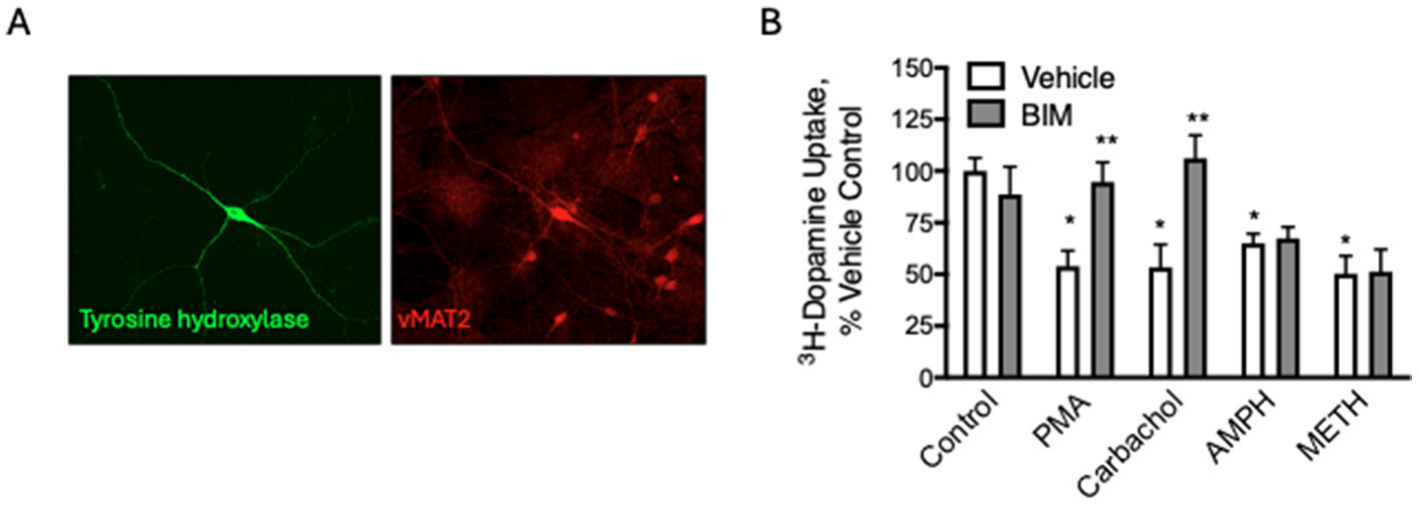
In primary midbrain cultures, PMA and carbachol-mediated loss of DAT function are PKC mediated while AMPH and METH-mediated redistribution are not. **(A)** Primary mid-brain cultures have dopamine neurons that express tyrosine hydroxylase (TH) and the vesicular monoamine transporter 2 (vMAT2). **(B)** Pre-treatment of these cultures with PMA (10 μM), carbachol (30 μM), amphetamine (10 μM, AMPH) and methamphetamine (10 μM METH) all decreased transport capacity of DAT (defined as GBR-12909 sensitive ^3^H-dopamine uptake). Bisindolylmaleimide I (BIM, 1 μM) inhibited the effects of PMA and carbachol, substantiating a PKC-mediated effect on DAT by these agonists. BIM did not alter the loss of DAT activity mediated by pretreatment with AMPH or METH indicating independent mechanisms of DAT internalization (*n* = 3).

The original version of this article has been updated.

